# Petechiae Post-dangling in Lower Extremity Free Tissue Transfer and Proposed Management

**DOI:** 10.7759/cureus.103105

**Published:** 2026-02-06

**Authors:** William West, Kristina T Buller, Mariel McArthur, Nicole K Le, Kristen Whalen, Jared Troy

**Affiliations:** 1 Morsani College of Medicine, University of South Florida, Tampa, USA; 2 Department of Plastic Surgery, University of South Florida, Tampa, USA

**Keywords:** anterolateral thigh (alt) flap, extremity reconstruction, flap dangle protocol, free alt flap, free flap monitoring, free flap reconstruction, petechiae

## Abstract

The anterolateral thigh (ALT) flap is often used by microsurgeons, with few reports of skin flap complications outside of partial flap necrosis. This study describes three unusual cases of flap dangle intolerance secondary to the development of immediate petechial hemorrhage of the skin paddle at the onset of dangle trials. Potential causes and management are described. A retrospective review was conducted of all free tissue transfers utilizing an ALT flap for lower extremity reconstruction performed by a single surgeon at a level 1 trauma center from October 2020 to May 2024. Patient risk factors were compared to identify potential causes of the dangle intolerance. Thirty-five free tissue transfers to reconstruct lower extremity defects with an ALT flap had a normal postoperative course until the initiation of the progressive dangle protocol. Evidence of petechial skin flap compromise was noted in three patients (8.6%) within five minutes of the initial dangle. These three flaps required up to a week of additional elevation to allow the full resolution of skin changes, only to have immediate return of the skin changes at the next trial of dangle. This process was repeated for up to six weeks before the flaps were able to tolerate a progressive dangle protocol without skin changes. No evidence of deep venous thrombosis (DVT) or thrombocytopenia was noted on Doppler ultrasound or laboratory work, respectively. In comparison to the control group, patient age, diabetes, hypertension, and smoking status were identified as potential risk factors. Delayed postoperative petechiae with progressive dangle protocols are an underreported issue that warrants more investigation. Increased patient age and comorbidities, including diabetes, hypertension, and current smoking, were identified in three patients who developed petechiae upon dangling. The reconstructions were completed successfully with a prolonged dangle protocol that others may utilize.

## Introduction

The anterolateral thigh (ALT) flap has been commonly used for reconstructive procedures since its initial introduction due to its versatility [1,2]. Complications of ALT flap use can be separated into donor site and recipient site, in addition to immediate and delayed postoperative complications. Potential immediate complications are microvascular revision and wound dehiscence, while potential delayed complications are wound healing difficulties and infection. Deep venous thrombosis (DVT) can be a devastating complication occurring immediately or in a delayed fashion [3].

In an endeavor to avoid postoperative complications in extremity reconstruction, patients are prescribed bed rest following the surgical procedure with the initiation of subsequent “dangling” protocols where the affected limb is placed in a gravity-dependent position for a progressively increasing period of time [4]. Dangling protocols vary by flap type, location, and surgeon preference [4-7]. Here, we describe three cases of the rapid onset of petechial hemorrhage of the skin paddle following the initiation of the dangling protocol after ALT flap free tissue transfer for lower extremity coverage. This complication has been described only once before in the literature [8]. We present our management and potential causes and risk factors based on a comparison with the other free ALT flaps for lower extremity reconstruction performed at our institution.

## Case presentation

Methods

All patients who underwent free transfer with an ALT flap for a lower extremity defect between October 2020 and May 2024 at a tertiary care hospital were reviewed retrospectively. Three cases are reviewed below in which the patient’s flap immediately developed petechiae upon the initiation of the dangle protocol, which required a considerable amount of time to resolve. The observed phenomenon occurred almost instantaneously, distinctly differing from the classic venous congestion typically seen at the onset of dangles. In contrast, normal venous congestion typically resolves within minutes or, at most, 1-2 days and does not present with petechiae. In addition to the three cases that developed petechiae upon the initiation of dangling, 32 other patients were identified for comparison to allow for the possible identification of petechia causes or risk factors. Data collected included patient demographics, comorbidities, injury etiology, wound size, the target of anastomoses, postoperative anticoagulation, and perioperative laboratory results and imaging.

Cases

Case 1

**Figure 1:**
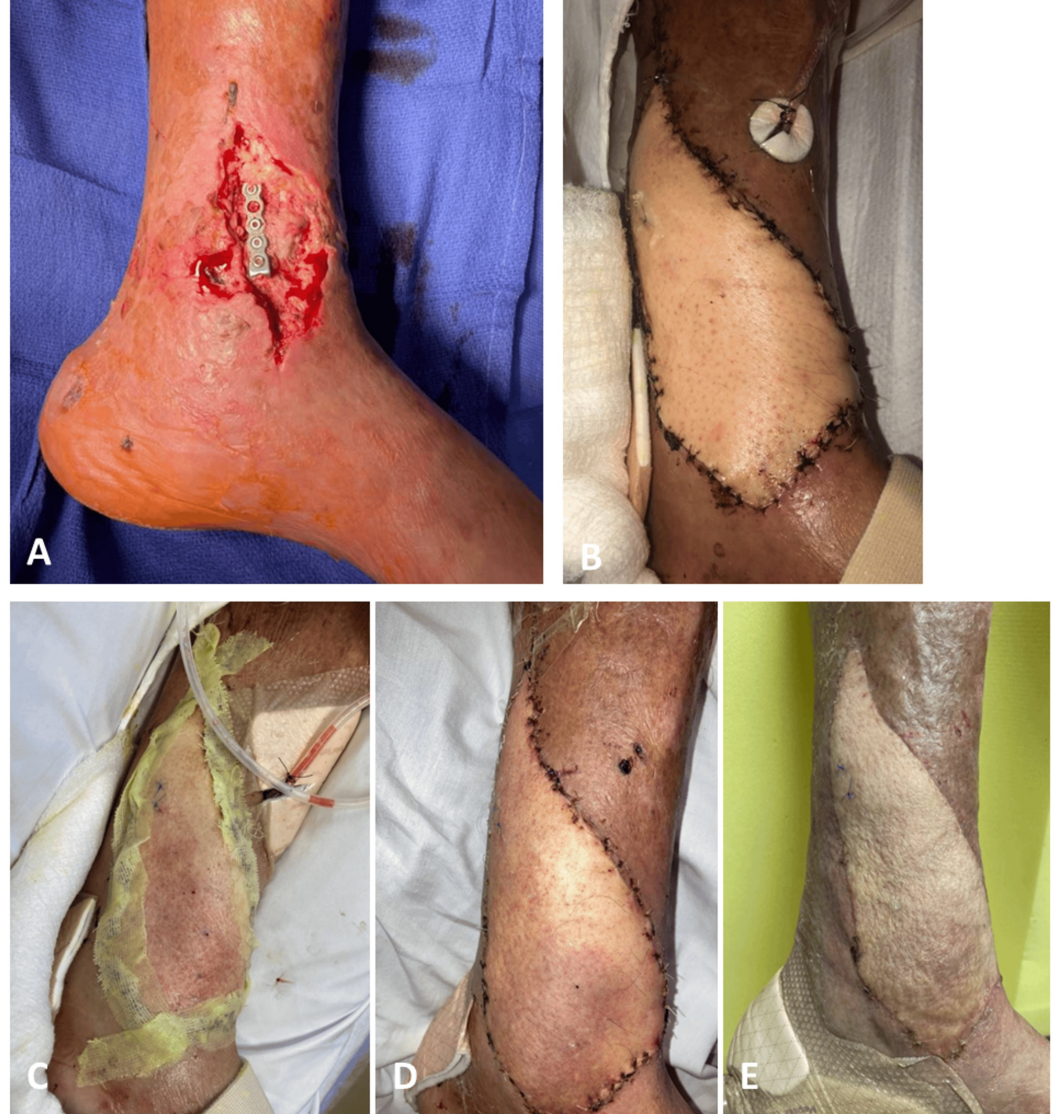
Case 1 (A) Three days before ALT flap coverage. (B) Postoperative day (POD) 4. (C) POD 6, when distal erythema was noticed the morning after the first dangle. (D) POD 28 post-dangle with petechiae noticed many times over the course of weeks. (E) POD 51, day of discharge ALT: anterolateral thigh

The first patient was a 63-year-old man who was involved in a motor vehicle accident and subsequently underwent external fixation, followed by open reduction and internal fixation for left pilon and fibular fractures (Figure 1). After developing wound breakdown and hardware exposure a month later, he underwent free tissue transfer utilizing the left ALT fasciocutaneous flap based on the descending branch of the lateral circumflex femoral artery and vein with end-to-end anastomosis to the posterior tibial artery and vein for a 20 × 8 cm wound on the medial left lower extremity. The patient was placed on strict bed rest with constant left leg elevation postoperatively. The patient began the dangle protocol on postoperative day (POD) 5. Flap color change with petechiae was noted immediately after the first five-minute dangle trial. Dangles were held until the skin paddle completely recovered to prevent compounding damage. On average, the flap required one week to fully recover. Additional dangle trials resulted in the same skin changes after a five-minute dangle trial. Ultimately, the flap required six weeks of dangle attempts before the petechiae were no longer present and the dangle interval could be progressively increased. Postoperatively, the patient remained on 325 mg of aspirin daily and 30 mg of subcutaneous enoxaparin twice a day for the remainder of his hospital stay. Workup for underlying causes of flap petechiae was unremarkable, including laboratory work negative for thrombocytopenia and prothrombin time (PT) and international normalized ratio (INR) within normal limits. Doppler ultrasound ruled out DVT.

Case 2

**Figure 2:**
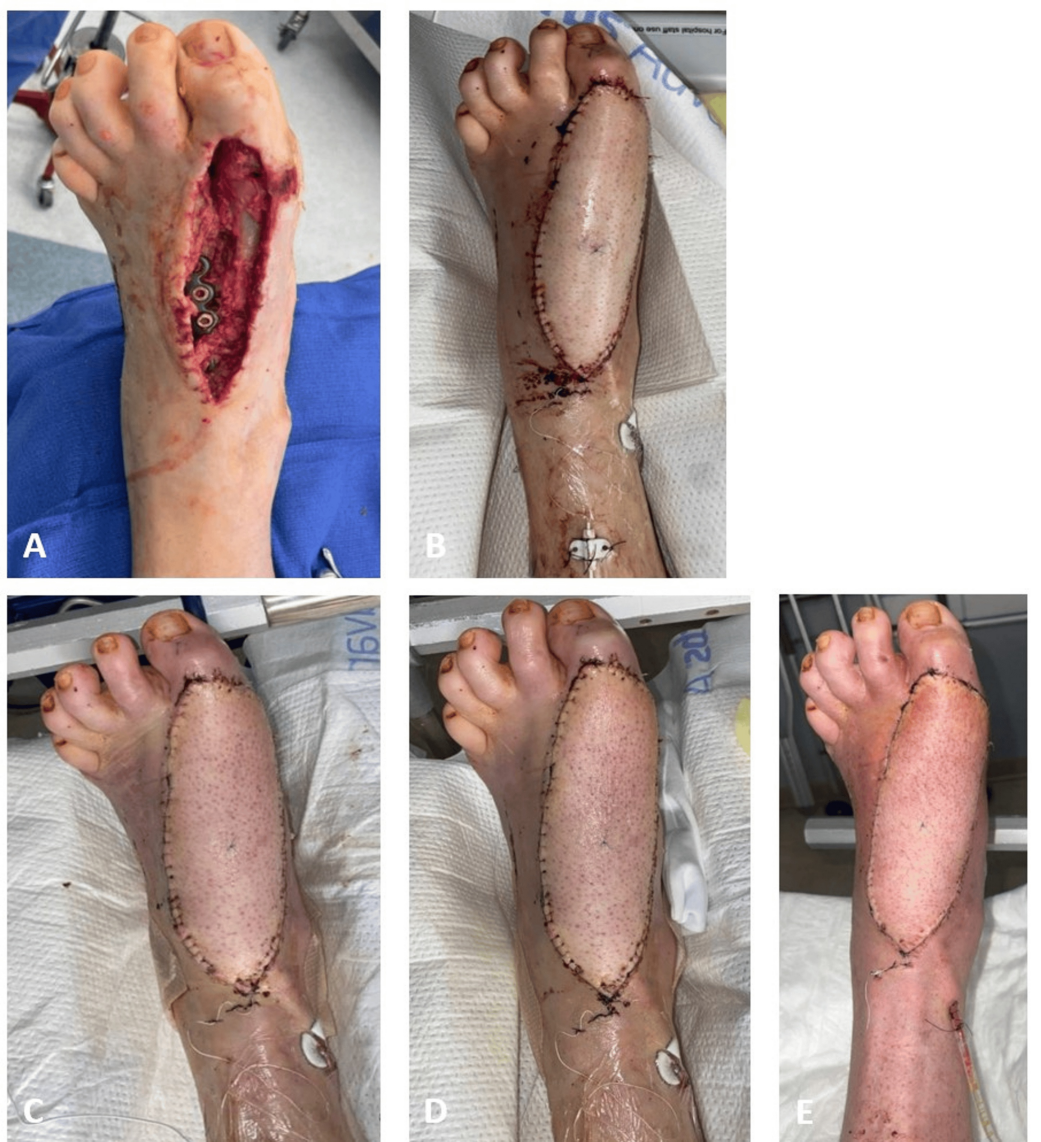
Case 2 (A) Four days before ALT flap coverage. (B) Postoperative day (POD) 2. (C) Congestion noted on the first day of dangling on POD 8. (D) POD 13 when petechiae were noted post-dangling. (E) Day of discharge on POD 34 ALT: anterolateral thigh

The second patient was a 39-year-old man with left Charcot foot with chronic midfoot dislocation secondary to type 2 diabetes mellitus (Figure 2). He underwent left midfoot fusion and Strayer procedure, subsequently developed an infection, and underwent multiple irrigation and debridement procedures by our orthopedic surgery colleagues. He subsequently underwent free tissue transfer of the left ALT fasciocutaneous flap based on the descending branch of the lateral femoral circumflex artery with end-to-end anastomosis to the dorsalis pedis artery and vein for a 5 × 15 cm left dorsal foot defect. The patient’s postoperative course was unremarkable, and the dangle protocol began on postoperative day 8 with the presence of petechiae after a five-minute dangle. Dangles were held until the complete resolution of the skin changes had occurred and required approximately one week between dangle trials. Ultimately, the flap required four weeks of dangle trial before there was no presence of petechiae. The patient was discharged home on postoperative day 34. Workup for underlying causes of flap petechiae was unremarkable, including laboratory work negative for thrombocytopenia and PT/INR within normal limits. Both computed tomography angiogram (CTA) of the bilateral lower extremities and venous Doppler ultrasound confirmed no DVT.

Case 3

**Figure 3:**
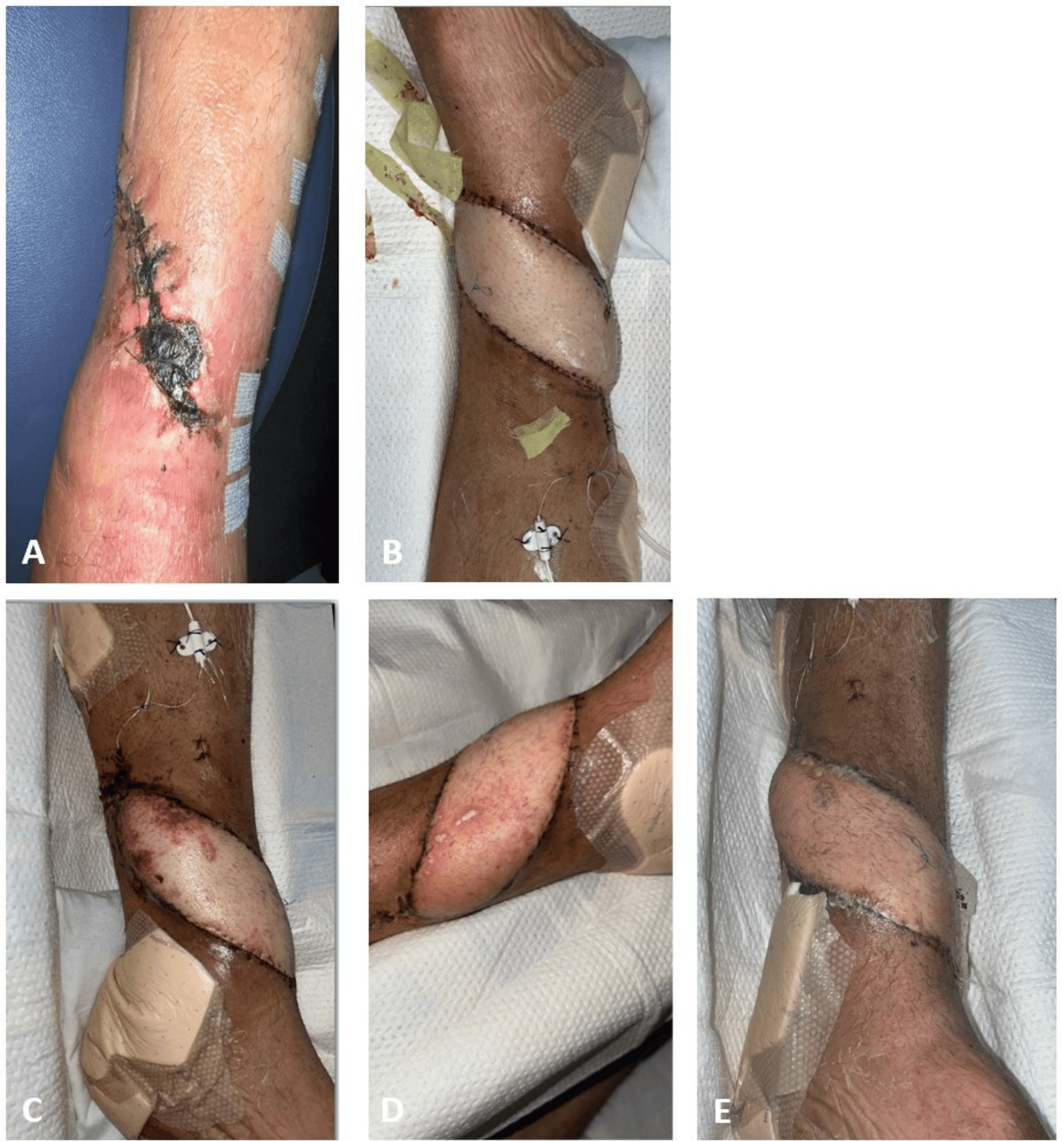
Case 3 (A) Twelve days before ALT flap coverage. (B) Postoperative day (POD) 3. (C) Ecchymoses and petechiae noted on the first day of dangling on POD 8. (D) POD 18, as petechiae were noticed again post-dangling. (E) Day of discharge on POD 29 ALT: anterolateral thigh

The third patient was a 52-year-old man who suffered a left open pilon fracture following a four-foot fall from a ladder (Figure 3). He developed hardware exposure following open reduction and internal fixation of the fracture. Therefore, a free tissue transfer of the right ALT fasciocutaneous flap based on the descending branch of the lateral femoral circumflex artery with end-to-side anastomosis to the dorsalis pedis artery and vein for a left dorsal lower extremity defect was performed. The dangle protocol began on POD 8 when ecchymosis and petechiae were first noted post-dangling. Dangles were held until POD 14 and progressed without complication until POD 18, when flap petechiae were again noticed. The dangle protocol was postponed for five more days and then restarted and completed without difficulty prior to discharge on POD 29. Workup for underlying causes of flap petechiae was unremarkable, including laboratory work negative for thrombocytopenia and PT/INR within normal limits. Preoperative computed tomography angiogram of the bilateral lower extremities revealed no DVT. Venous Doppler ultrasound of the upper extremities during the postoperative period revealed right subclavian DVT, but there was no thrombosis in the bilateral lower extremities.

Results

The three patients who developed petechiae had an average hospital length of almost twice as long as the 32 patients without petechiae upon dangling (45 ± 8 versus 23 ± 1 days). The average age of the three petechia patients was 51.3 ± 6.9 years compared to 39.0 ± 2.4 years in the patients who tolerated dangling. All three petechia patients had medical comorbidities, including diabetes (100%) and hypertension (66.7%). Gender, race, smoking status, flap size, anastomosis target vessel, and time from surgery to first dangle did not appear to differ between the two patients who developed petechiae and the 19 who did not (Table 1). The patient in Case 1 did have a history of coronary artery disease with percutaneous coronary intervention, as well as hyperlipidemia, while the patients in Cases 2 and 3 did not.

**Table 1:** Comorbidities and outcome data

Variable	No Petechiae (n = 32)	Petechiae (n = 3)
Age and years	39.0 ± 2.4	51.3 ± 6.9
Gender		
Female	3 (9.3%)	0 (0.0%)
Male	29 (90.6%)	3 (100.0%)
Race/ethnicity		
Non-Hispanic White	18 (56.3%)	2 (66.7%)
Hispanic	6 (18.8%)	1 (33.3%)
Non-Hispanic Black	8 (25.0%)	0 (0.0%)
Smoking status		
Never	10 (31.3%)	1 (33.3%)
Former	7 (21.9%)	0 (0.0%)
Current	15 (46.9%)	2 (66.7%)
Diabetes mellitus	5 (15.6%)	3 (100.0%)
Hypertension	8 (25.0%)	2 (66.7%)
Hyperlipidemia	3 (9.4%)	1 (33.3%)
Peripheral vascular disease	0 (0.0%)	0 (0.0%)
Target vessels		
Anterior tibial	12 (37.5%)	0 (0.0%)
Posterior tibial	19 (59.4%)	2 (66.7%)
Others	1 (3.1%)	1 (33.3%)
Flap size, cm^2^	168 ± 15	118 ± 43
Time from injury to reconstruction, days	301 ± 171	42 ± 5
Time from reconstruction to first dangle, days	6 ± 1	7 ± 1
Hospital length of stay, days	23 ± 1	45 ± 8
Hospital length of stay after first dangle, days	9 ± 1	31 ± 8

## Discussion

One previous study described similar petechial hemorrhaging upon dangling initiation in a free groin flap based on the superficial epigastric vessels anastomosed to the anterior tibial vessels. On venogram, the veins were varicose, so they hypothesized that the incompetent valves allowed for pressure buildup in the capillary bed with petechial bleeding. They were unsure why the petechiae did not appear again with mobilization after two more weeks of bed rest [8]. In our two cases, it is possible that the veins were varicose, given that no venogram was performed. It is also possible that diabetes, age, and hypertension were risk factors for petechia development with dangling.

Diabetes has been previously noted as a risk factor for the development of petechiae after skin trauma from heat or cold, potentially due to a combination of microangiopathy and neuropathy, as these conditions impede the flow of oxygen and metabolites, as well as the ability of the body to alter local blood flow, respectively [9]. Diabetes, hypertension, and age have all been noted as risk factors for the development of the Rumpel-Leede phenomenon, in which a petechial rash develops as a result of acute dermal capillary rupture. Diabetic microangiopathy increases capillary fragility, and hypertension increases venous pressure [10]. Age further exacerbates the capillary fragility [11,12].

All three patients began tolerating dangling without petechiae after allowing for sufficient time for the complete resolution of petechiae from the first attempted dangle and instituting a protocol in which the flap was wrapped with an elastic compression bandage during the dangle. Ultimately, the dangle protocol was held between one and 12 days at various times due to petechia development. It is hard to make definitive conclusions about how long providers should hold dangles and when they should accept full dangle tolerance if they encounter flap petechiae in their practice; however, a case-by-case approach should be taken with clinical evaluation. The only other case report on this topic reported that they held their dangle protocol for two weeks with subsequent full dangle tolerance [8]. We recommend holding further dangles until the skin paddle has complete resolution of skin changes to prevent compounding injury to the flap and risking further flap complications. This took approximately 5-7 days. During this time, a workup with venous Doppler ultrasound is reasonable. When dangles are attempted, pre- and post-dangle flap checks should be conducted, along with compression during and in between dangles.

This study has several limitations when trying to determine the root cause of petechial development. There are only three cases, making statistical analysis between the cases and the control group impossible. Additionally, there is no standard workup for this condition, which further exacerbates the difficulty of more clearly delineating the cause of petechial bleeding.

## Conclusions

We believe that the development of skin paddle petechiae upon dangling is an underreported issue. Additional work is needed to identify the underlying cause so that high-risk patients can be identified preoperatively and underlying factors can possibly be corrected to prevent the increased length of stay. Providers should be aware of this complication, and we recommend that management include holding the dangle protocol until the resolution of petechiae with additional workup of potential causes, including platelet levels via complete blood count, platelet function studies, PT/INR, venous Doppler ultrasound, and CTA of the lower extremities. Compression should be used when restarting the dangle protocol.
